# Sirtuin Inhibitors Are Broadly Antiviral against Arboviruses

**DOI:** 10.1128/mBio.01446-19

**Published:** 2019-07-09

**Authors:** Brent A. Hackett, Mark Dittmar, Elisha Segrist, Nathan Pittenger, Julie To, Trevor Griesman, Beth Gordesky-Gold, David C. Schultz, Sara Cherry

**Affiliations:** a Department of Microbiology, University of Pennsylvania, Philadelphia, Pennsylvania, USA; b High-Throughput Screening Core, University of Pennsylvania, Philadelphia, Pennsylvania, USA; University of Colorado School of Medicine; University of Massachusetts Medical School; Icahn School of Medicine at Mount Sinai

**Keywords:** alphavirus, arbovirus, bunyavirus, flavivirus, sirtinol, sirtuin

## Abstract

Arthropod-borne viruses are diverse pathogens and are often associated with human disease. These viruses span multiple genera, including flaviviruses, alphaviruses, and bunyaviruses. In a high-throughput drug screen, we found that tenovin-1 was antiviral against the flaviviruses Zika virus and dengue virus. Tenovin-1 is a sirtuin inhibitor, and here we found that inhibition of sirtuins, but not inhibition of the related histone deacetylases, is potently antiviral against diverse arboviruses. Sirtuin inhibitors block infection of arboviruses in multiple human cell types. We found that sirtuin inhibitors arrest infection downstream of entry but that they do so at an early step, preventing the accumulation of viral RNA and protein. However, sirtuin inhibitors had no impact on the replication of flaviviral replicons, suggesting a defect in the establishment of replication. Consistent with this, we found that sirtuin inhibitors impacted double-stranded RNA (dsRNA) accumulation during flaviviral infection. Since these viruses infect vector insects, we also tested whether sirtuin inhibitors impacted infection of adult flies and found that these inhibitors blocked infection; therefore, they target highly conserved facets of replication. Taken together, these results suggest that sirtuin inhibitors represent a new class of potent host-targeting antivirals.

## Abstract (precis)

Arthropod-borne viruses are diverse pathogens and are associated with human disease. Through high-throughput drug screening, we found that sirtuin inhibitors are potently antiviral against diverse arboviruses, including flaviviruses such as West Nile virus, bunyaviruses such as Rift Valley fever virus, and alphaviruses such as chikungunya virus. Sirtuin inhibitors block infection of these viruses in multiple human cell types. Moreover, we found that sirtuin inhibitors arrest infection downstream of entry but that they do so at an early step, preventing the accumulation of viral RNA and protein. Since these viruses infect vector insects, we also tested whether sirtuin inhibitors impacted infection of adult flies and found that these inhibitors blocked infection; therefore, they target highly conserved facets of replication. Taken together, these results suggest that sirtuin inhibitors represent a new class of potent host-targeting antivirals.

## INTRODUCTION

Arthropod-borne viruses (arboviruses) are transmitted to humans by insect vectors, typically, mosquitoes, and are emerging and reemerging globally. There are three major groups of arboviruses that infect humans: flaviviruses, alphaviruses, and bunyaviruses (1). Flaviviruses are the most widespread group and are enveloped positive-sense RNA viruses that include dengue virus (DENV), which infects hundreds of millions of people yearly; Zika virus (ZIKV), which recently emerged in the Americas; yellow fever virus (YFV), which causes high mortality in the Americas and Africa; and West Nile virus (WNV), which became endemic in the New World in the last 2 decades (2). Alphaviruses are another medically relevant group of viruses that are also enveloped positive-stranded RNA viruses. These include chikungunya virus (CHIKV), which emerged in the last decade, and Sindbis virus (SINV), which is the prototypical alphavirus (3). Bunyaviruses, the third major group of arboviruses, are enveloped trisegmented negative-sense viruses that include many important human pathogens such as Rift Valley fever virus (RVFV), which has a devastating impact on livestock in Africa, and La Crosse virus (LACV), which is responsible for high rates of encephalitis in children in the United States (4). There are no approved antiviral treatments for any of these arboviruses and a dearth of vaccines.

We previously performed a small-molecule screen to identify antivirals active against ZIKV (5). We identified and validated three drugs that were antiviral against ZIKV across three different cell types: nanchangmycin, mycophenolic acid, and tenovin-1. Nanchangmycin and mycophenolic acid inhibit viral entry and RNA replication, respectively (5). It is unknown how tenovin-1 controls viral infection. Tenovin-1 has been shown to have activity against sirtuins (SIRTs), which are an evolutionarily conserved family of lysine deacetylases (KDACs) (6). The family of SIRTs is comprised of seven KDACs that require the cofactor NAD and that are homologous to the yeast protein *Sir2p* (7). SIRTs are present in nuclear and cytoplasmic compartments (8). Also, several of these proteins have been previously shown to impact a broad range of viral pathogens (9–12). In some cases, SIRTs promote infection, while in other cases, SIRTs restrict infection (9, 10, 13–19). Whether sirtuin inhibitors impact arbovirus infection has not yet been explored.

Here we found that sirtuin inhibitors are potently antiviral against a panel of diverse arboviruses. Tenovin-1 is antiviral against diverse arboviruses from three major groups of pathogens, namely, flaviviruses, alphaviruses, and bunyaviruses. Moreover, additional sirtuin inhibitors are antiviral against these viruses in multiple cell types. And they can be very potent; treatment with these inhibitors can result in multilog drops in infection levels. Mechanistic studies have suggested that sirtiuns are required postentry. Moreover, we observed a defect in the early phases of replication, as there was a decrease in double-stranded RNA (dsRNA) accumulation, representing one of the earliest steps in replication. We also found that this dependency is evolutionarily conserved, as adult flies treated with sirtuin inhibitors show decreased infection, and this is exacerbated in SIRT1 or SIRT2 mutants. Taking the data together, our studies suggest that the availability of SIRT inhibitors of greater potency and bioavailability would allow the development of a new class of potent and broad-spectrum antiviral agents.

## RESULTS

### Tenovin-1 is broadly antiviral.

**FIG 1:**
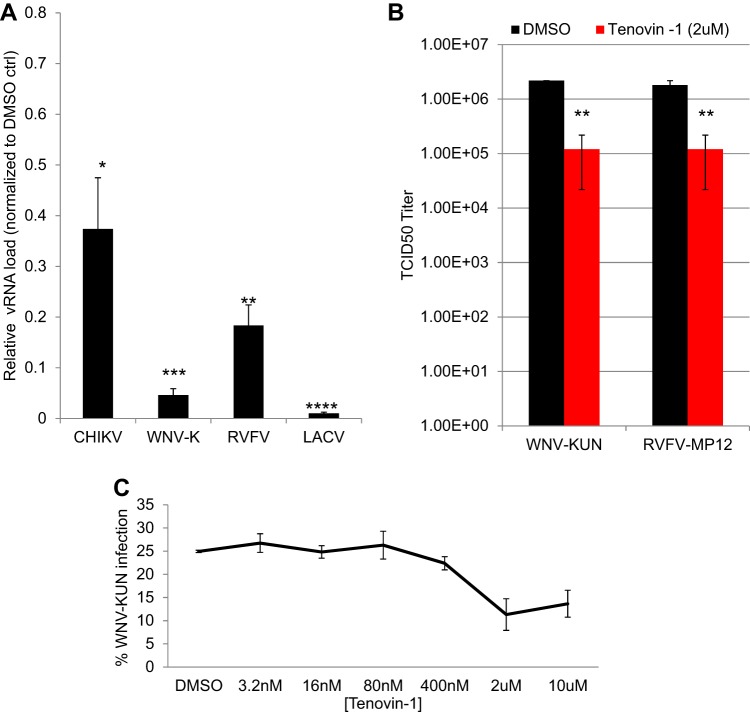
Tenovin-1 is broadly antiviral against arboviruses. (A) U2OS cells were infected with the indicated virus (MOI of 0.5, 24 hpi) and at 2.5 h postinoculation, vehicle control (DMSO ctrl) or 2 μM tenovin-1 was added. Total cellular RNA was collected and analyzed by RT-qPCR. Data represent means ± standard errors of the means (SEM), *n* = 3; *, *P* < 0.05; **, *P* < 0.01; ***, *P* < 0.001; ****, *P* < 0.0001 (Student's *t* test). vRNA, viral RNA. (B) TCID_50_ analysis of WNV-Kunjin and RVFV MP12 in U2OS cells, comparing vehicle control (DMSO) to 2 μM tenovin-1, added at 2.5 hpi. Data represent means ± SEM. *n* = 3; **, *P* < 0.01 (Student's *t* test). (C) U2OS cells were infected with WNV-Kunjin (MOI of 0.5, 24 hpi) for 2.5 h prior to addition of tenovin-1 at the indicated concentration. Percent infection was quantified by automated microscopy; data representing means ± SEM of results from two experiments are shown.

Our previous studies showed that tenovin-1 is antiviral against Zika virus (5). We first sought to examine the breadth of this inhibition using phylogenetically distinct viruses. We observed that tenovin-1 (2 μM) significantly reduces viral load in U2OS cells during infection with the flavivirus West Nile virus (WNV; Kunjin strain), with the alphavirus Chikungunya virus (CHIKV; 181/25), or with disparate bunyaviruses, including Rift Valley fever virus (RVFV; MP12) and La Crosse virus (LACV; original), as measured by quantitative reverse transcription-PCR (RT-qPCR) (Fig. 1A). We also determined the magnitude of the inhibition by measuring the impact of tenovin-1 on viral titers and found that tenovin-1 treatment led to a 1 log reduction of levels of WNV (Kunjin) and RVFV (MP12) (Fig. 1B). And we found that the antiviral activity was dose dependent and nontoxic by the use of a microscopy-based assay (Fig. 1C; see also Fig. S1A in the supplemental material).

10.1128/mBio.01446-19.1 FIG S1 Sirtuin inhibitors are nontoxic. (A to D) U2OS cells were treated with (A) tenovin-1 or (B) sirtinol or (C) EX-527 or (D) AGK2 at the indicated concentrations, and cell numbers were determined by microscopy at 24 h posttreatment. Download FIG S1, PDF file, 0.1 MB. Copyright © 2019 Hackett et al. 2019 Hackett et al. This content is distributed under the terms of the Creative Commons Attribution 4.0 International license.

### Sirtuin inhibitors are antiviral.

**FIG 2:**
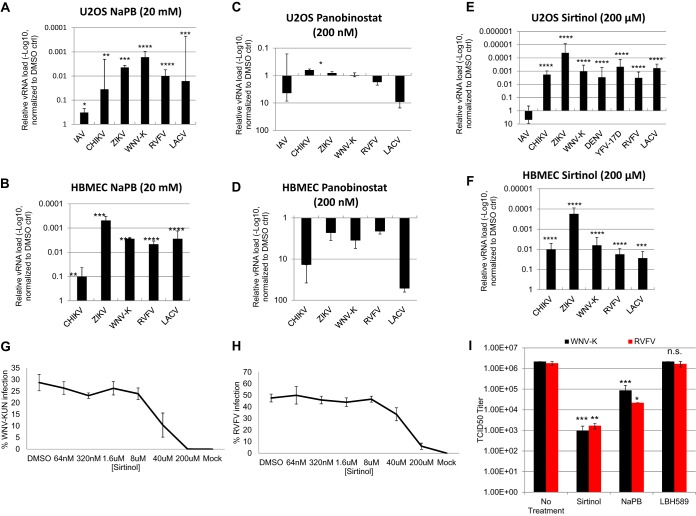
Sirtuin inhibitors, but not other HDAC inhibitors, are antiviral against diverse arboviruses. U2OS cells (A, C, and E) or HBMECs (B, D, and F) were infected with the indicated virus (MOI of 0.5 [ZIKV MOI of 2.5], 24 h). At 2.5 hpi, vehicle control or (A and B) 20 mM NaPB or (C and D) 200 nM panobinostat or (E and F) 200 μM sirtinol was added. Total cellular RNA was collected and analyzed by RT-qPCR. Data represent means ± SEM. *n* = 3; *, *P* < 0.05; **, *P* < 0.01; ***, *P* < 0.001; ****, *P* < 0.0001 (Student's *t* test). (G and H) U2OS cells were infected with (G) WNV-Kunjin or (H) RVFV-MP12 (MOI of 0.5, 24 hpi) for 2.5 h prior to addition of sirtinol at the indicated concentration. Percent infection was quantified by automated microscopy; means ± SEM of results from two experiments are shown. (I) TCID_50_ analysis of WNV-K and RVFV MP12 in U2OS cells was performed with the indicated compound added at 2.5 hpi. Data represent means ± SEM. *n* = 3; n.s., not significant; **, *P* < 0.01 (Student's *t* test).

While tenovin-1 is known to inhibit SIRTs, it may also impact other targets (6). We set out to determine if the antiviral effect of tenovin-1 was due to SIRT inhibition. First, we tested whether the pan-KDAC inhibitor sodium phenylbutyrate (NaPB) impacted infection of this panel of arboviruses. In addition, we tested influenza A virus (IAV) as previous studies suggested that sirtuins are antiviral against this virus (10). We found that treatment with NaPB reduced viral replication of CHIKV, ZIKV, WNV, RVFV, and LACV by more than 1 log in both U2OS cells and human brain microvascular endothelial cells (HBMECs) (Fig. 2A and B). In contrast, we observed only a modest reduction in IAV (PR8) infection in U2OS cells.

To address which class(es) of KDACs may be involved, we tested panobinostat (LBH589), which inhibits all classes of KDACs except SIRTs (20). And we found that treatment with this inhibitor did not attenuate infection of these viruses but rather that there was a trend toward increasing levels of infection in both U2OS cells and HBMECs (Fig. 2C and D).

Next, we tested sirtinol, which is a specific SIRT1 and SIRT2 inhibitor and does not inhibit class I and class II KDACs. We found that sirtinol potently inhibited CHIKV, ZIKV, WNV, RVFV, and LACV by more than 2 logs in U2OS cells and more than 1 log in HBMECs as measured by RT-qPCR (Fig. 2E and F). Indeed, ZIKV was the most sensitive, displaying 4-log reductions in infection (Fig. 2E). As was previously observed, sirtinol treatment led to increased infection by IAV, suggesting a distinct interaction (Fig. 2E). Using a microscopy-based assay, we found that the sirtinol inhibition of WNV and RVFV was dose dependent and nontoxic (Fig. 2G; see also Fig. S1B). The sirtinol 50% inhibitory concentration (IC_50_) against WNV and RVFV was ∼40μM.

We confirmed the antiviral role of sirtinol against WNV and RVFV by measuring viral titers. We found that panobinostat had no impact on WNV or RVFV titers whereas sodium phenylbutyrate and sirtinol reduced viral titers in U2OS cells (Fig. 2I). Indeed, sirtinol reduced titers by approximately 3 logs.

### Inhibition of SIRT1 or SIRT2 is not sufficient to block arboviral infection.

**FIG 3:**
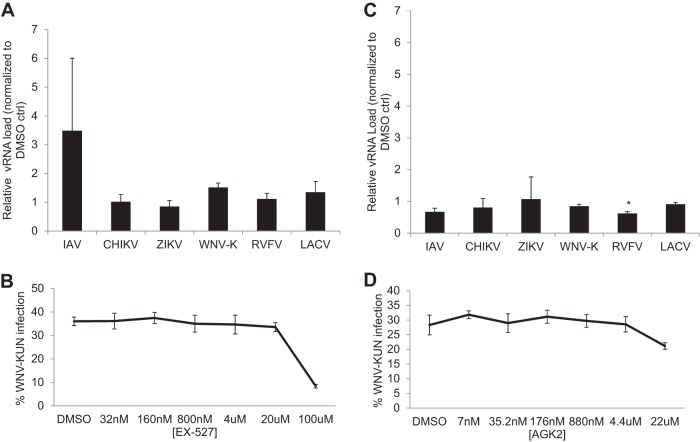
Selective inhibition of SIRT1 or SIRT2 is not antiviral. (A and C) U2OS cells were infected with the indicated virus (MOI of 0.5 [ZIKV MOI 2.5], 24 h). At 2.5 hpi, vehicle or (A) 100 nM SIRT1 inhibitor EX527 or (C) 22 μM SIRT2 inhibitor AGK2 was added. Total cellular RNA was collected and analyzed by RT-qPCR. Data represent means ± SEM. *n* = 3; *, *P* < 0.05 (Student's *t* test). (B and D) U2OS cells were infected with WNV-Kunjin (MOI of 0.5, 24 hpi) for 2.5 h prior to addition of (B) EX527 or (D) AGK2 at the indicated concentration. Percent infection was quantified by automated microscopy; means ± SEM of results from three experiments are shown.

Sirtinol inhibits both SIRT1 and SIRT2 (21). Therefore, we set out to determine if selective inhibition of SIRT1 or SIRT2 alone could phenocopy sirtinol treatment. We first tested selisistat (EX527), which targets SIRT1 with an IC_50_ of 100 nM and displays >200-fold selectivity against SIRT2. We used 100 nM EX527, which selectively targets SIRT1 (22). We found that treatment with EX527 had no impact on arboviral infection in U2OS cells (Fig. 3A). And dose-response analysis of WNV-Kunjin (WNV-K) revealed that, indeed, EX527 was active only at the highest concentration (100 μM), which is known to inhibit additional SIRTs, and had no toxicity (Fig. 3; see also Fig. S1C).

Next, we tested the selective SIRT2 inhibitor AGK2, which has an IC_50_ of 3.5 μM and inhibits SIRT1 at >10-fold-higher concentrations. We found that AGK2 at a high concentration (22 μM) did not reduce infection by any virus by more than 40% (Fig. 3C). We also performed a dose-response analysis for WNV-Kunjin and observed no reduction in infection or toxicity (Fig. 3D; see also Fig. S1D). Since inhibition of SIRT1 or SIRT2 was not sufficient, we also tested the combination of EX527 and AGK2 to determine whether that combination could block infection. We found that the combination did not block WNV-Kunjin infection (Fig. S2). Therefore, the results indicated that inhibition of SIRT1 and SIRT2 is not sufficient to block viral infection but suggested that, instead, inhibition of multiple SIRTs is required to inhibit arboviral infection.

10.1128/mBio.01446-19.2 FIG S2 Combination of AGK2 and EX-527 does not impact WNV infection. U2OS cells were infected with WNV-Kunjin (MOI of 0.5, 24 h). At 2.5 hpi, vehicle or 100 nM SIRT1 inhibitor EX527 or 3.5 μM SIRT2 inhibitor AGK2 or the combination was added. Total cellular RNA was collected and analyzed by RT-qPCR. Data represent means ± SEM. *n* = 3. Download FIG S2, PDF file, 0.03 MB. Copyright © 2019 Hackett et al. 2019 Hackett et al. This content is distributed under the terms of the Creative Commons Attribution 4.0 International license.

### The requirement for SIRTs in arboviral infection is deeply conserved.

**FIG 4:**
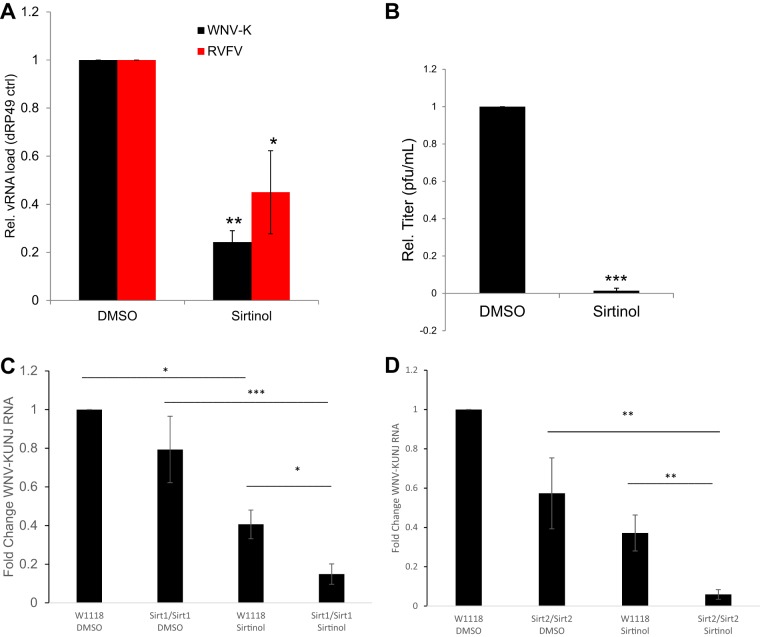
Sirtinol blocks viral infection at the organismal level in adult flies. (A) Adult flies were treated with vehicle or sirtinol and infected with either WNV-Kunjin or RVFV-MP12. At 7 dpi, groups of 15 flies were processed for RT-qPCR analysis. Data represent means ± SEM. *n* = 3; *, *P* < 0.05; ****, *P* < 0.0001 (Student's *t* test). (B) Experiments were performed as described for panel A; adult flies were treated with vehicle or sirtinol and infected with WNV-Kunjin. Groups of 5 flies were processed 7 dpi and subjected to plaque assay normalized to control. Data represent means ± SEM. *n* = 3. *, *P* < 0.05 (Student's *t* test). (C and D) Adult flies of the indicated genotypes were treated with vehicle or sirtinol and infected with WNV-Kunjin as described for panel A. At 7 dpi, groups of 15 flies were processed for RT-qPCR analysis. Data represent means ± SEM. *n* = 3; *, *P* < 0.05; **, *P* < 0.01; ***, *P* < 0.001 (one-way analysis of variance [ANOVA] with multiple comparisons).

SIRTs are evolutionarily conserved from yeast to humans. Moreover, the arboviruses are transmitted between vertebrates and insect vectors and thus have to replicate in cells of these divergent organisms. We have previously used *Drosophila* as a model for insect vectors and have infected flies with WNV-Kunjin and RVFV-MP12 (23, 24). Insects encode 5 SIRTs, including SIRT1 and SIRT2 orthologs (25). Moreover, sirtinol has been used in *Drosophila* to inhibit SIRT function (26, 27). Therefore, we tested whether sirtinol would be antiviral against these arboviruses at the organismal level in adult flies. We treated adult female flies with vehicle or sirtinol and subsequently challenged the animals with WNV-Kunjin or RVFV-MP12. Groups of 15 flies were collected 7 days postinfection (7 dpi), and viral infection was monitored by RT-qPCR across three independent experiments. We observed a significant decrease in infection upon treatment with sirtinol (Fig. 4A). Next, we determined the viral titer in samples from flies challenged with WNV-Kunjin upon treatment with sirtinol. Again, we found a significant reduction in viral titers; whereas the samples from the control flies had an average of 318 PFU/ml, the samples from the sirtinol-treated flies had 17 PFU/ml, representing a reduction of more than 1 log (Fig. 4B). Taken together, these data suggest that inhibition of SIRTs can block arboviral infection across host phyla.

To further implicate SIRTs in the control of infection, we obtained flies harboring a CRISPR deletion in *Drosophila* SIRT1 (dSIRT1) (2a-7-11) or dSIRT2 (5B-2-35). We challenged control flies or mutants with WNV-Kunjin along with vehicle or sirtinol. We monitored infection by RT-qPCR and found that SIRT1 mutants displayed reduced infection and that the level was further reduced upon treatment with sirtinol (Fig. 4C). SIRT2 mutants did not display significantly reduced infection; however, sirtinol treatment led to a strong reduction in infection of SIRT2 mutant flies (Fig. 4D). Taken together, these data suggest that there is a redundant requirement for at least SIRT1 and SIRT2 in WNV infection.

### Sirtinol treatment impacts postentry steps.

**FIG 5:**
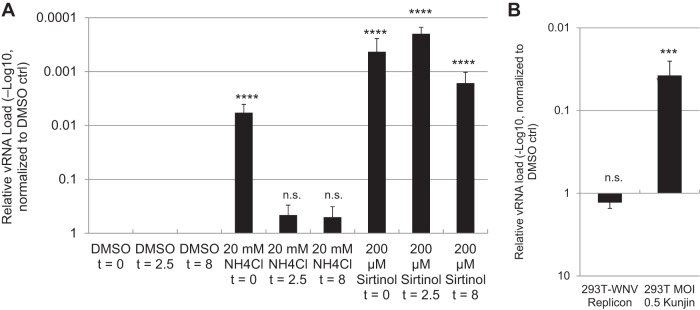
Sirtinol inhibits postentry steps prior to release but not established infection. (A) U2OS cells were treated with vehicle (DMSO), ammonium chloride (NH_4_Cl), or sirtinol at the indicated time relative to WNV-Kunjin (MOI of 0.5, 24 h). At 2.5 hpi, NH_4_Cl was added to all wells to block viral spread. Total cellular RNA was collected and analyzed by RT-qPCR. Data represent means ± SEM. *n* = 3; ****, *P* < 0.0001 (Student's *t* test compared to control). (B) 293T cells were infected with WNV-Kunjin (MOI of 0.5, 24 h), or 293T cells harboring WNV-Kunjin replicons were treated with vehicle (DMSO) or sirtinol (200 μM) for 24 h, and total cellular RNA was analyzed by RT-qPCR. Data represent means ± SEM. *n* = 3; ***, *P* < 0.001 (Student's *t* test).

We set out to determine whether sirtinol impacts viral entry or early steps in the viral life cycle. Therefore, we performed time-of-addition studies using WNV infection as the model. Here we added drugs at the same time as infection, at 2.5 h postinfection, or at 8 h postinfection. We added ammonium chloride to samples at 2.5 h postinfection to block spread. We found that while ammonium chloride blocked infection when added at the same time as WNV, it lost activity when added at 2.5 h postinfection (a time postentry). In contrast, sirtinol remained active even when added 8 h postinfection, a time when replication had just begun to launch as measured by RT-qPCR (Fig. 5A). Sirtinol was more active than ammonium chloride, reducing infection by more than 3 logs when added at the time of infection or at 2.5 h postinfection, and began to decline in potency when added at 8 h postinfection (hpi).

### Sirtinol treatment does not impact established replication.

These data suggest that sirtinol was inhibiting an early step in replication, as viral RNA and viral protein production were lost from the infected cells. Moreover, sirtinol was working at a step postentry. In addition, we observed the replication defect in single-round infections, suggesting that the inhibition was independent of assembly and egress.

This led us to test whether sirtinol would be active against flavivirus replicons (WNV-Kunjin) that express green fluorescent protein (GFP) in place of the structural proteins (28). These replicons are stably expressed and replicate in 293T cells. They constitutively have replication foci and produce viral RNA and proteins but cannot form new particles as they are lacking structural proteins. We first verified that sirtinol was antiviral against WNV-Kunjin in 293T cells and found that treatment with sirtinol significantly reduced WNV replication by RT-qPCR (Fig. 5B). Subsequently, we treated 293T cells carrying the replicons with sirtinol and found that this treatment did not reduce viral RNA loads at 24 h posttreatment, a time when the replication inhibitor MK-0608 attenuated infection (Fig. 5B; see also Fig. S3). These data suggest that cells with replication intermediates preestablished are refractory to treatment.

10.1128/mBio.01446-19.3 FIG S3 Replication inhibitor MK-0608 attenuates infection and replicon replication. 293T cells were infected with WNV-Kunjin (MOI of 0.5, 24 h), or 293T cells harboring WNV-Kunjin replicons were treated with vehicle (DMSO) or MK-0608 (10μM) for 24 h, and total cellular RNA levels were analyzed by RT-qPCR. Data represent means ± SEM. *n* = 3; *, *P* < 0.05; **, *P* < 0.01 (Student’s *t* test). Download FIG S3, PDF file, 0.1 MB. Copyright © 2019 Hackett et al. 2019 Hackett et al. This content is distributed under the terms of the Creative Commons Attribution 4.0 International license.

### Sirtinol treatment impacts the establishment of replication foci.

**FIG 6:**
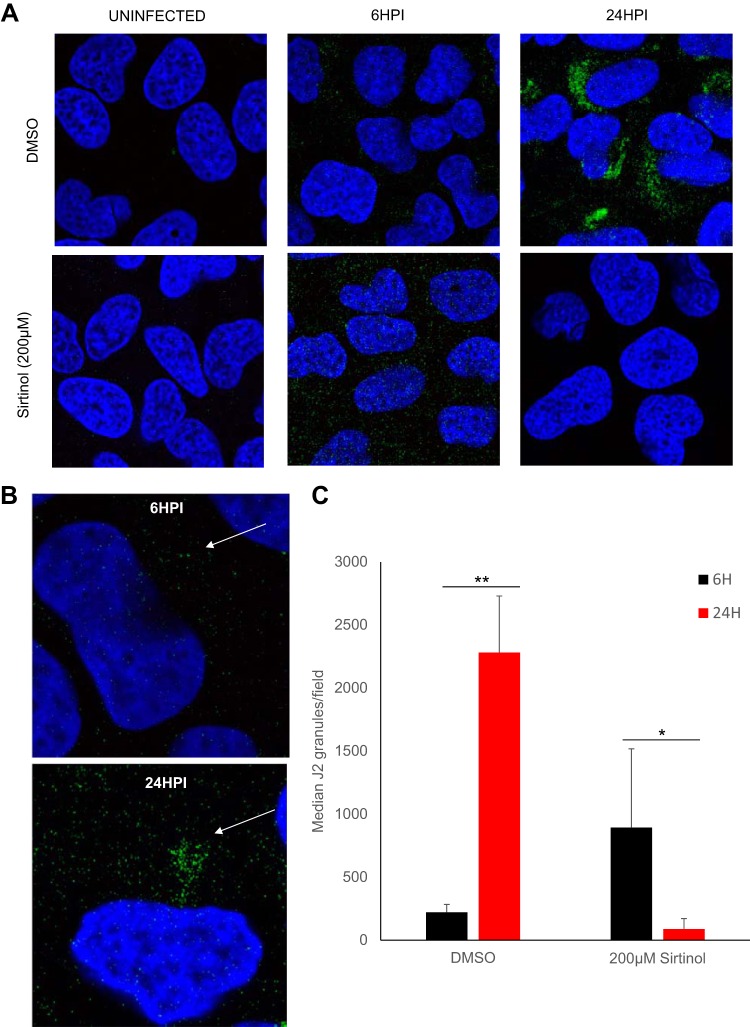
Sirtinol treatment arrests dsRNA replication. (A and B) U2OS cells were left uninfected or infected with WNV-Kunjin (MOI of 1), and vehicle (DMSO) or sirtinol (200 μl) was added at 2 hpi. Following fixation at the indicated time point postinfection, cells were stained for dsRNA replication intermediates (anti-J2, green) and total nuclei (DAPI [4′,6-diamidino-2-phenylindole], blue). (B) Images representative of results of three independent experiments; representative higher-magnification images from DMSO controls are shown. (C) Quantification of granules shown in panel A. Data represent means ± SEM. *n* = 3; *, *P* < 0.05; **, *P* < 0.01 (Student's *t* test).

One of the earliest steps in the replication cycle postentry is the establishment of replication foci where double-stranded RNA (dsRNA) accumulates. Therefore, we hypothesized that this step might be targeted by sirtinol. Using an antibody that recognizes dsRNA (J2), we monitored the accumulation of replication intermediates during WNV-Kunjin infection. We found that these foci became visible at 6 hpi but that they were small (Fig. 6). Furthermore, these foci became larger as replication complexes were established and were found to have increased in abundance more than 100-fold by 24 hpi (Fig. 6). Next, we determined whether sirtinol impacted dsRNA focus formation and accumulation. We found that sirtinol-treated cells displayed similar levels of small dsRNA foci at 6 hpi (Fig. 6). Moreover, we found that the levels did not increase with time but that they were lost by 24 hpi. These data suggest that establishment and maintenance of replication complexes are compromised by sirtinol treatment blocking viral replication at an early step postentry but at a step bypassed by replicon-containing cells, as they had already established robust replication.

## DISCUSSION

Arboviruses are a diverse group of RNA viruses that depend on host factors for their replication and that in some cases hijack the same cellular genes and pathways. For example, many arboviruses are dependent on clathrin-mediated endocytosis for entry. Thus, host factors and small-molecule inhibitors that impact this entry pathway also impact viral infection by diverse arboviruses and do so across hosts. Indeed, we performed a high-throughput screen for antivirals active against ZIKV across three distinct cell types and identified three drugs that were broadly active: mycophenolic acid, nanchangmycin, and tenovin-1. Mycophenolic acid is a nucleoside analog that inhibits RNA-dependent RNA polymerase activity across diverse RNA viruses in insects and mammals. Nanchangmycin is an entry inhibitor that inhibits diverse viruses that use clathrin-mediated endocytosis for entry, including these arboviruses. Therefore, we set out to explore tenovin-1 as a new antiviral that may potentially impact diverse viral families.

Tenovin-1 was previously found to be an inhibitor of sirtuins, in particular, of SIRT1 and SIRT2. Therefore, we tested whether additional SIRT inhibitors were active against ZIKV, as well as a larger panel of arthropod-borne viruses. Sirtuins are a subgroup of KDACs that also include histone deacetylases (HDACs); thus, we first tested the pan-KDAC inhibitor NaPB. In addition, we tested the HDAC inhibitor panobinostat and the more specific SIRT1/SIRT2 (SIRT1/2) inhibitor sirtinol. We found that the pan-KDAC inhibitor and sirtinol were antiviral against a panel of arboviruses, including the flaviviruses ZIKV, WNV, and DENV, the alphavirus CHIKV, and the bunyaviruses RVFV and LACV, while the HDAC inhibitor panobinostat was not antiviral against these viruses. Moreover, we found that these drugs were active in multiple cell types. Interestingly, and consistent with previous literature, inhibition of sirtuins did not attenuate infection of IAV, suggesting that the sirtuin inhibitors show some specificity for these arboviruses.

Sirtuins (SIRTs) are an evolutionarily conserved set of NAD^+^-dependent enzymes that are primarily known as lysine deacetylases. While their functions have not been fully elucidated, it is clear that SIRTs impact a wide range of cellular pathways, driven at least in part by their subcellular localizations. In addition, data suggest that these proteins can have alternative enzymatic activities, making their potential roles even more complex. Our findings suggest that inhibition of at least SIRT1 and SIRT2 is required for antiviral activity, since inhibition of either SIRT and of the combination of the specific SIRT1/2 inhibitors did not block viral infection.

Since arboviruses infect arthropods as part of their life cycle, and because sirtuins are highly conserved, we also tested whether sirtinol would impact infection of insects. Therefore, we treated adult flies with sirtinol and monitored infection by two arboviruses, WNV and RVFV. And we found that, indeed, sirtinol attenuated infection in adult flies. We took advantage of the fact that the dSIRT1 and dSIRT2 mutants are viable to determine if these SIRTs are required for infection. We found that loss of SIRT1 or SIRT2 was not sufficient to block infection; however, sirtinol treatment of either mutant led to strong reductions in infection. Taken together, our data suggest that inhibition of multiple SIRTs is required to block infection of diverse viruses. Given that these drugs are active at concentrations in the high micromolar range, further development of these therapeutics is necessary to move them into the clinic.

Previous studies have shown that sirtuins can have antiviral or proviral effects, depending on the virus (9–12). For example, SIRT inhibition increased influenza A virus titers and activated Kaposi's sarcoma-associated herpesvirus (KSHV) (10, 19). In contrast, other studies have found that inhibition of SIRTs can result in antiviral activity against diverse viruses. For example, sirtinol inhibits hepatitis A virus replication, human cytomegalovirus (CMV) infection, and hepatitis B virus infection (11, 12, 14–18). In general, the mechanisms involved have remained unclear.

Mechanistically, we explored the step in the virus life cycle impacted by sirtuin inhibition. We found that sirtinol blocks infection downstream of entry, as the drug remained active after we added the inhibitor to cells postentry. Indeed, sirtinol blocks all of the arboviruses downstream of entry. This block occurs early during the replication cycle since the initial accumulation of viral proteins and RNAs is blocked across arboviruses as observed with our microscopy-based assay as well as RT-qPCR-based assays. Next, we took advantage of cell lines that harbor flavivirus replicons. These replicons encode the proteins required for translation and replication but cannot enter or exit the cells. We found that replicon cells could not be inhibited by sirtinol. This suggested that a very early step in the establishment of a replication niche might have been blocked, since these cells had already established this compartment pretreatment. Therefore, we monitored the accumulation of dsRNA during the early steps in the establishment of infection and found that dsRNA accumulated early, in an aberrant form, and that the dsRNAs were unable to increase in level to meet the needs of replication but instead were lost. Taken together, these data suggest that remodeling of cellular membranes by lysine deacetylation is required for WNV replication and, potentially, for replication of the other arboviruses.

Taking the data together, we suggest that SIRT inhibitors potentially represent a new class of host-directed antiviral therapeutics. The development of SIRT inhibitors with greater potency and bioactivity may be transformative.

## MATERIALS AND METHODS

### Cells.

U2OS cells (ATCC HTB-96) and 293T cells (ATCC CRL-3216) were cultured in Dulbecco's modified Eagle medium (DMEM; Gibco) supplemented with 10% fetal bovine serum, 1% penicillin/streptomycin (Gibco), and 1× nonessential amino acids (Gibco). Cells were incubated at 37°C with 5% carbon dioxide. 293T replicon cells (a gift of T. Pierson, NIAID) were cultured in 10 μg/ml blasticidin S prior to use in assays. HBMECs were cultured in RPMI 1640 medium supplemented with 10% fetal bovine serum (FBS), 10% NuSerum, minimum essential medium (MEM), vitamins, nonessential amino acids, sodium pyruvate, and antibiotics (29).

### Virus stocks.

ZIKV (Mex2-81), WNV (Kunjin), and yellow fever virus (YFV 17D) were obtained from R. Tesh (The World Reference Center for Emerging Viruses and Arboviruses [WRCEVA] at The University of Texas Medical Branch [UTMB]) and from M. Diamond (Washington University). CHIKV-mKate (181/25) was obtained from M. Heise (University of North Carolina). LACV (original) was obtained from S. Soldan (Wistar), and RVFV (MP12) was from R. Doms (University of Pennsylvania). IAV (PR8) was propagated in eggs and obtained from S. Hensley (University of Pennsylvania). ZIKV, WNV, YFV, and CHIKV were propagated in C636, and titers were determined on Vero cells. LACV and RVFV were grown in BHK cells as previously described (30).

### Antibodies.

Monoclonal antibody to flavivirus envelope (4G2) and RVFV glycoprotein (4D4) were obtained from M. Diamond (Washington University) and C. Schmaljohn (USAMRIID), respectively. J2 monoclonal antibody was a gift of Carolyn Coyne (University of Pittsburgh).

### Drugs.

Drugs for this study were purchased as follows: tenovin-1 was purchased from Tocris Bioscience (catalog no. 336510), sirtinol from Cayman Chemical (catalog no. 10523), sodium phenylbutyrate from Sigma (catalog no. P21005), panobinostat from Cayman Chemical (catalog no. 13280), selisistat/EX527 from Cayman Chemical (catalog no. 10009798), and AGK2 from Cayman Chemical (catalog no. 13145). Drugs were resuspended in dimethyl sulfoxide (DMSO), except for NaPB, which was resuspended in water. Drugs were diluted in culture media for use.

### Viral analysis by RT-qPCR.

U2OS cells or HBMECs were treated with the indicated virus and drugs and processed for total RNA using TRIzol reagent (Invitrogen) and an RNA Clean and Concentrate kit (Zymo Research). cDNA was generated from random hexamers to prime reverse transcription reactions using Moloney murine leukemia virus (M-MLV) reverse transcriptase. Quantitative PCR (qPCR) was performed with the cDNA using Power SYBR green PCR master mix and a QuantStudio 6 Flex RT-PCR system (Applied Biosystems). Primer sequences used in qPCR reactions are listed in Table S1 in the supplemental material. Reactions were initially run at an initial 95°C for 5 min and then in 40 cycles of 95°C for 20 s, 52°C for 30 s, and 72°C for 30 s. For drug assays, relative viral copy numbers were generated by normalization to cells treated with DMSO.

10.1128/mBio.01446-19.4 TABLE S1 Primers used. Download Table S1, PDF file, 0.1 MB. Copyright © 2019 Hackett et al. 2019 Hackett et al. This content is distributed under the terms of the Creative Commons Attribution 4.0 International license.

### Time of addition.

U2OS cells were pretreated with drugs 1 h prior to infection or at the indicated times postinfection. In addition, 20 mM ammonium chloride was added at 2.5 hpi and the cells were processed at 24 hpi.

### Dose-response analyses.

U2OS cells were inoculated with the indicated virus at a multiplicity of infection (MOI) of 2.5 for 24 h in 96-well format. At 2.5 hpi, culture medium was replaced with media containing 5-fold dilutions of specified drug. Cells were then processed for automated imaging. Formaldehyde-fixed cells were processed, stained for nuclei (Hoechst 333342) or viral antigen (virus-specific antibody), and imaged by automated microscopy (using at least three wells per condition for at least three sites per well; ImageXpressMicro; ×10 magnification), and the images were subjected to automated image analysis using MetaXpress software as previously described (31).

### Viral titering (TCID_50_ analysis).

10-fold serial dilutions of WNV-K or RVFV in DMEM complete growth medium were used to infect seeded U2OS cells in a 96-well plate. At 24 h postinfection, cells were fixed in 4% formaldehyde, immunostained for viral envelope proteins, and imaged by automated immunofluorescence microscopy (ImageXpressMicro; ×10). Values corresponding to 50% tissue culture infective doses (TCID_50_) were calculated according to the method of Reed and Muench (32).

### Fly infections.

Female wild-type (WT) flies or flies of the indicated genotypes (WT [w1118], dSIRT1 [2a-7-11], and dSIRT2 [5B-2-35]) (7 to 10 days old) were obtained from Bloomington and were injected with ∼50 nl of DMSO or 50 mM sirtinol diluted 1:10 in WNV-Kunjin (∼10^7^ PFU/ml) or RVFV-MP12 (∼10^7^ PFU/ml). At 7 days postinfection, groups of flies were crushed in TRIzol for total RNA purification or were crushed in Schneider’s media for viral titer analysis by TCID_50_.

### Confocal microscopy.

Cells on glass coverslips were treated as described above, fixed with 4% formaldehyde, and washed twice with phosphate-buffered saline (PBS) before blocking was performed for 30 min in PBS containing 1% Triton X-100, 2% bovine serum albumin (BSA), and 0.02% sodium azide. Primary antibody dilutions were made in same block buffer, and cells were incubated overnight at 4°C. Following two PBS washes, secondary antibody treatment was done for 1 h at 4°C. Coverslips were then mounted to glass slides with 15% (vol/vol) glycerol–PBS. Images were taken with a Leica CTR 6500 confocal microscope, and data were quantified using the granularity module in the MetaXpress software suite (Molecular Devices).
