# Supplementary figures and images for: Sirtuin Inhibitors Are Broadly Antiviral against Arboviruses
Source: mBio. 2019 Jul 9;10(4):e01446-19. doi: 10.1128/mBio.01446-19 (PMC6747726; doi:10.1128/mBio.01446-19)

**A**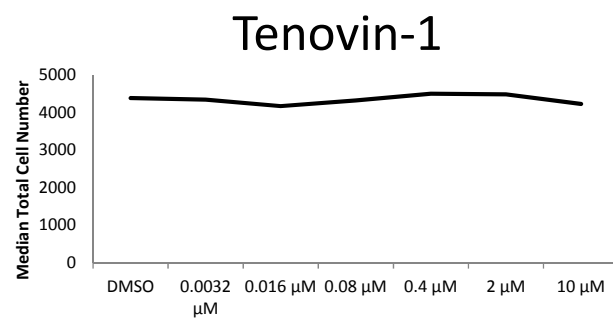**B**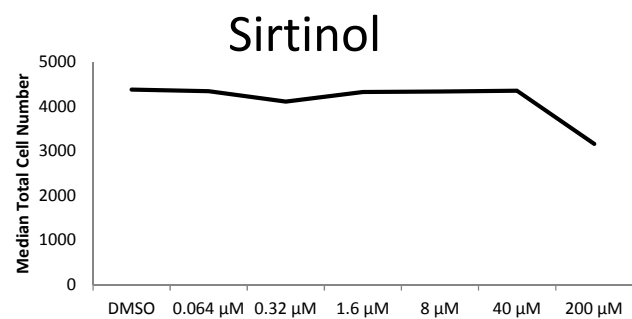**C**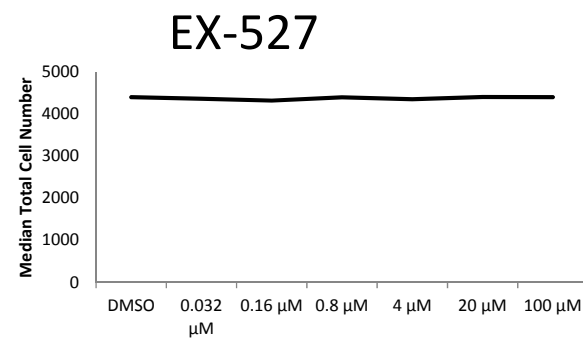**D**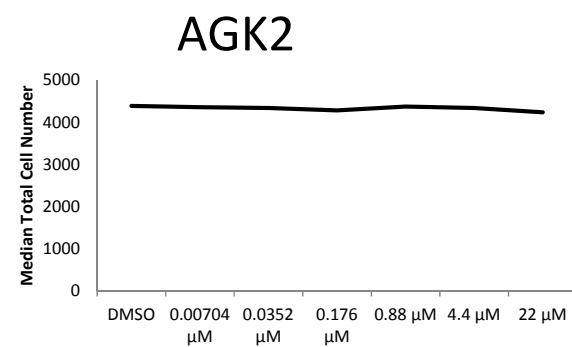

Supplement: FIG S1 [file mBio.01446-19-sf001.pdf]

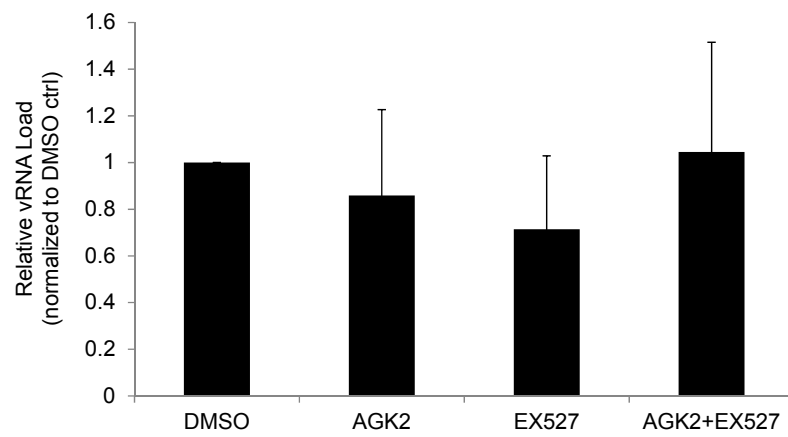

Supplement: FIG S2 [file mBio.01446-19-sf002.pdf]

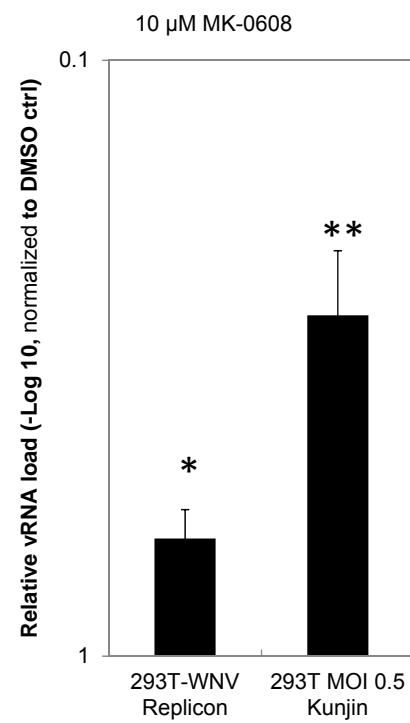

Supplement: FIG S3 [file mBio.01446-19-sf003.pdf]
