# Supplementary material for: Sirtuin Inhibitors Are Broadly Antiviral against Arboviruses
Source: mBio. 2019 Jul 9;10(4):e01446-19. doi: 10.1128/mBio.01446-19 (PMC6747726; doi:10.1128/mBio.01446-19)
Supplement: TABLE S1 [file mBio.01446-19-st001.pdf]

Table S1: Primers used for RT-qPCR

Target	Forward Primer	Reverse Primer
WNV	CCTGTGTGAGCTGACAAACTTAGT	GCGTTTTAGCATATTGACAGCC
RVFV	CAAGCAGTGGACCGCAATGAGA	GGGCTTGTTGCCACGAGTTAGA
LACV	GCTGAGTCTAATGGTGTAGGATG	TGGTCAGCGGGTAGAATTTG
CHIKV	GGCAGTGGTCCCAGATAATTCAAG	ACTGTCTAGATCCACCCCATACATG
IAV	TTAGGATTTGTGTTCACGCTCACCG	CCAGCCATTTGCTCCATAGCCTTG
YFV	TGGCATATTCCAGTCAACCTTCT	GAAGCCCAAGATGGAATCAACT
DENV2	TGAGGACTACATGGGCTCTG	AAACCTCCCTGGATTTCCTT
ZIKV	GTGTCATACTGTGGGCCTTG	AATATTCCAGGCAGGGTCTG
GAPDH	ACCAAATCCGTTGACTCCGACCTT	TCGACAGTCAGCCGCATCTTCTTT
